# Effect of Er on Microstructure and Mechanical Properties of 5052 Aluminum Alloy with Big Width-To-Thickness Ratio

**DOI:** 10.3390/ma13030568

**Published:** 2020-01-24

**Authors:** Xinwei She, Xianquan Jiang, Bao Qi, Kang Chen

**Affiliations:** 1 School of Materials and Energy, Southwest University, Tiansheng Road 2, Beibei District, Chongqing 400715, China; 13983073429@163.com (X.S.); qibao17830909705@163.com (B.Q.); chenkang@cqut.edu.cn (K.C.); 2 Advanced Materials Research Center, Chongqing Academy of Science and Technology, Yangliu Road 2, Chongqing 401123, China

**Keywords:** aluminum alloy, Er, microstructure, mechanical properties

## Abstract

The effect of Er on microstructure and mechanical properties of the 5052 aluminum alloy with a big width-to-thickness ratio was investigated by a metallurgical microscope, scanning electron microscope and tensile testing machine. The results showed that the precipitates were slightly refined after Er addition and Al_3_Fe was transformed into Al_6_Fe and AlEr with/without a small amount of Fe or Si. The effect of Er on grain refinement was related to its content. When Er content was lower or higher than 0.4%, the grain would coarsen. Homogenization could refine the grain by controlling Er content and distribution in the Al matrix. Long time homogenization at high temperature would significantly reduce the strength of the 5052 aluminum alloy and 5052 aluminum alloys with low Er content, but help to improve the plasticity of those with high Er content. The ultimate tensile strength, yield strength and elongation of the as-cast 5052 aluminum alloy were 197 MPa, 117 MPa and 22.5% respectively. The strength was the highest, when Er content was 0.4 wt. % and the elongation was the best at 0.1 wt. % Er content.

## 1. Introduction

With the continuous innovation of aviation, aerospace and other high-techs, people have put forward higher and higher requirements for the performance of aluminum alloys in recent years [1,2,3]. Aluminum alloys are developing towards great strength, high toughness and excellent corrosion resistance, and alloying or micro-alloying is one of the most effective methods [4,5,6,7]. A large number of literatures show that the addition of rare earth elements to aluminum alloys can play a positive role in optimizing the microstructure and mechanical properties. Among them, Sc is the most prominent, and others like Er, La, Ce, Yb, etc. also have a good development potential [8,9,10,11,12,13,14,15].

5XXX series aluminum alloys are widely used in aerospace, automobile and ship due to their low density, high specific strength, good corrosion resistance and easy processing [16,17,18,19]. The 5052 aluminum alloy is one of the most typical and commonly used aluminum alloys in the 5XXX series only containing Mg as a strengthening element. It is easy to crack in plastic deformation, which greatly limits its application space [20,21]. At present, a 5052 aluminum alloy ingot with a big width-to-thickness ratio is prone to edge crack because of the serious segregation along the width direction, which makes it difficult to meet the requirements of large deformation. Therefore, it seems a new research topic in the 5052 aluminum alloy to improve its strength and reduce its cracking tendency.

In this paper, the microstructure and mechanical properties of the 5052 aluminum alloy with a big width-to-thickness ratio in as-cast, homogenized and cold-rolled states are studied by adding trace Er into a 5052 aluminum alloy, in order to provide a theoretical basis for improving the deformability of (ultra) wide 5052 aluminum alloy plates.

## 2. Materials and Methods

The materials are 5052 aluminum alloys with/without different Er content cast by a metal model. The chemical composition is shown in Table 1.

**Figure 1:**
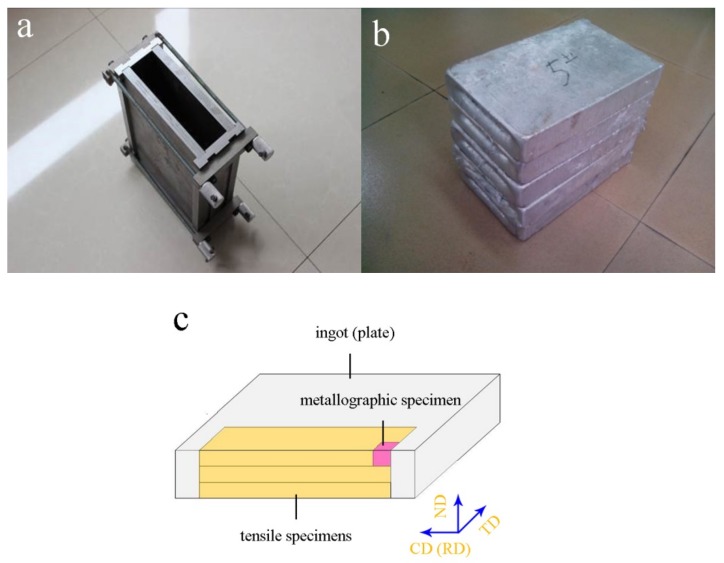
Ingot and sampling method: (**a**) metal model assembled by mechanical connection; (**b**) ingots cast at 700–720 °C and (**c**) sampling method, mainly wire cutting.

Figure 1 shows the ingot prepared by a metal model and the sampling method. A set of metal models made of heat-resistant die steel was designed to simulate the forming process and microstructure characteristics of (ultra) wide aluminum alloy ingots. The wall and the base with a thickness of 15 mm were assembled by a mechanical connection (Figure 1a). First, an energy saving industrial furnace (SG 2-12-10, Dongtai, China) was used to heat alloy raw materials to 750 °C for melting. Then, heat preservation and standing for 30 min after mixing evenly. Finally, the casting temperature was controlled at 700–720 °C, and the ingot size was 300 mm × 200 mm × 50 mm (length × width × thickness; Figure 1b). A laboratory electric furnace (SRX 2-12-12, Chongqing, China) was used for homogenization with a temperature of 550 °C and a holding time of 20 h. The ingot was cooled with furnace cooling. After homogenization, the ingot was milled and its dimension before rolling should be controlled at 220 mm × 180 mm × 40 mm. The four-rollers non reversing rolling mill (Φ 350, Wuxi, China) was used for hot rolling and cold rolling of the ingot with a total reduction of 36 mm. Three sample plates were cut along the casting direction at the edge of the ingot or rolling plate, and processed into tensile specimens according to GB/T 228-2002 standard. The ultimate tensile strength (UTS), yield strength (YS) and elongation (EL) were measured by tensile testing machine (CMT4503, Shanghai, China) at room temperature with a tensile speed of 2 mm/min and their average values were calculated. Metallographic specimen with a size of 10 mm × 10 mm × 10 mm was cut from the undeformed end of tensile specimens for mechanical grinding and electrolytic polishing. Metallurgical microscope (ZEISS, Oberkochen, Germany) was used to observe the second phase and grain (Figure 1c). Scanning electron microscope (JEOL, Tokyo, Japan) was applied to observe the microstructure of metallographic specimen and fracture morphology of tensile specimen. Meanwhile, energy dispersive spectrometer (X-Max, Oxford, UK) was taken to identify phase composition and element distribution.

## 3. Results and Discussion

### 3.1. Microstructure

**Figure 2:**
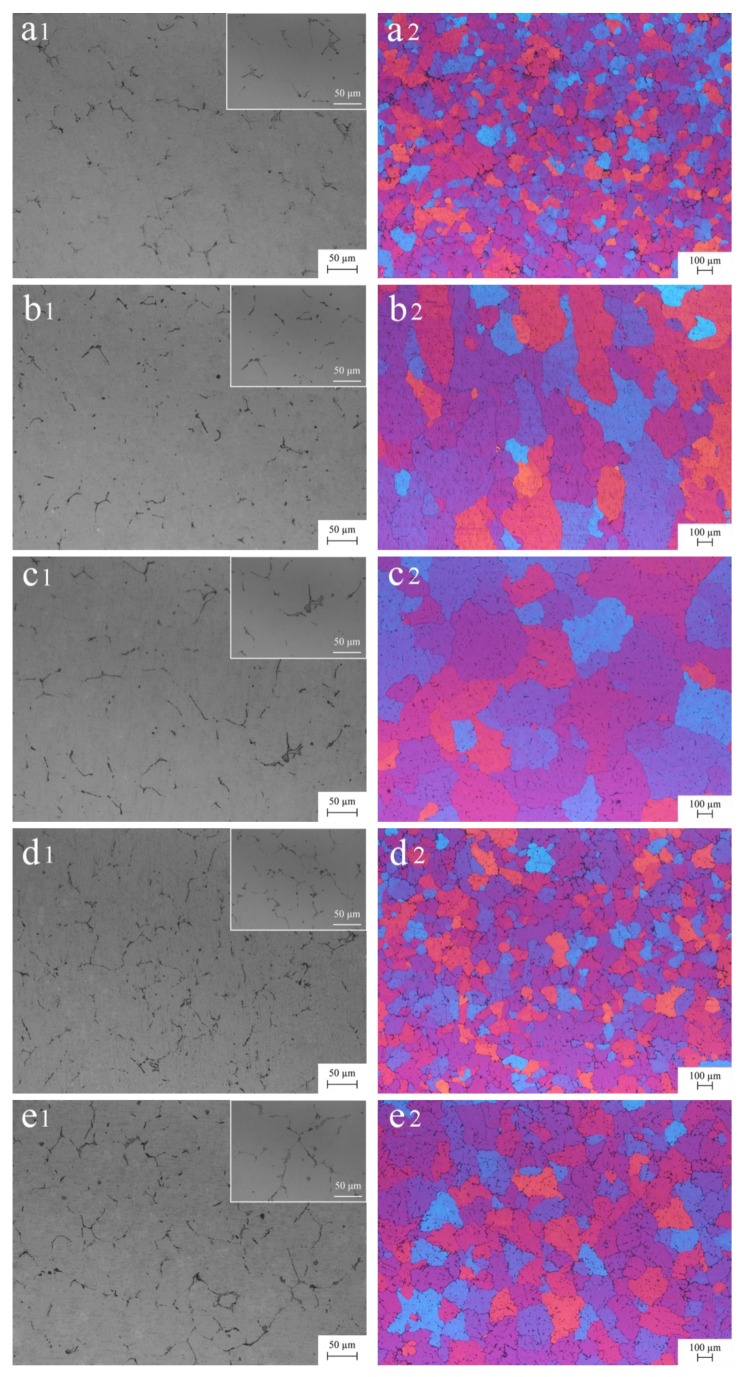
Metallographic structure of as-cast 5052 aluminum alloys with embedded graphs to reflect the local characteristics on the top right of images: (**a1**,**a2**) A0 without Er; (**b1**,**b2**) A1 with 0.1% Er; (**c1**,**c2**) A2 with 0.2% Er; (**d1**,**d2**) A3 with 0.4% Er and (**e1**,**e2**) A4 with 0.8% Er.

Figure 2 shows the metallographic structure of as-cast 5052 aluminum alloys. A0 was mainly composed of α-Al and the second phase distributed along the grain boundary. The second phase with a dimension of about 50 μm had two main morphologies, acicular and Chinese script (Figure 2a1). After adding Er, the second phase was slightly refined. With the increase of Er content, the number of the second phase increased. When Er content was lower than 0.4%, the second phase mostly presented acicular, and when it was higher than 0.4%, the Chinese script phase was obviously increased (Figure 2b1–e1). Furthermore, when Er content was 0.2%, the second phase suddenly grew up to 80 μm, when it increased to 0.4%, the second phase abruptly became fine, approximately 30 μm, and when further increasing, the second phase began to grow up again and the impurities increased (Figure 2c1–e1). The addition of an appropriate amount of Er was conducive to refining the microstructure and the increase of Er content could promote the transformation of the second phase from acicular to Chinese script [22]. It could be seen that the transition point was 0.4%. The grain of A0 was nearly equiaxed and its size distribution was uneven, about 116 μm (Figure 2a2). This was mainly due to the faster cooling rate and the larger temperature gradient at the edge of the ingot, resulting in a different growth rate of grain. After the addition of Er, the grain size had an obvious change. When Er content was less than 0.4%, the grain presented coarsened and its size was more than 400 μm. Moreover, the grain tended to be equiaxed with the increase of Er content (Figure 2b2,c2). When Er content was 0.4%, the grain was rapidly refined to 159 μm and its size distribution was also inhomogeneous, and when it reached 0.8%, the grain exceptionally grew again with a mean size of 243 μm, accompanied by dendrite formation (Figure 2d2,e2). It could be inferred that the refining effect of Er on grain was related to its content.

**Figure 3:**
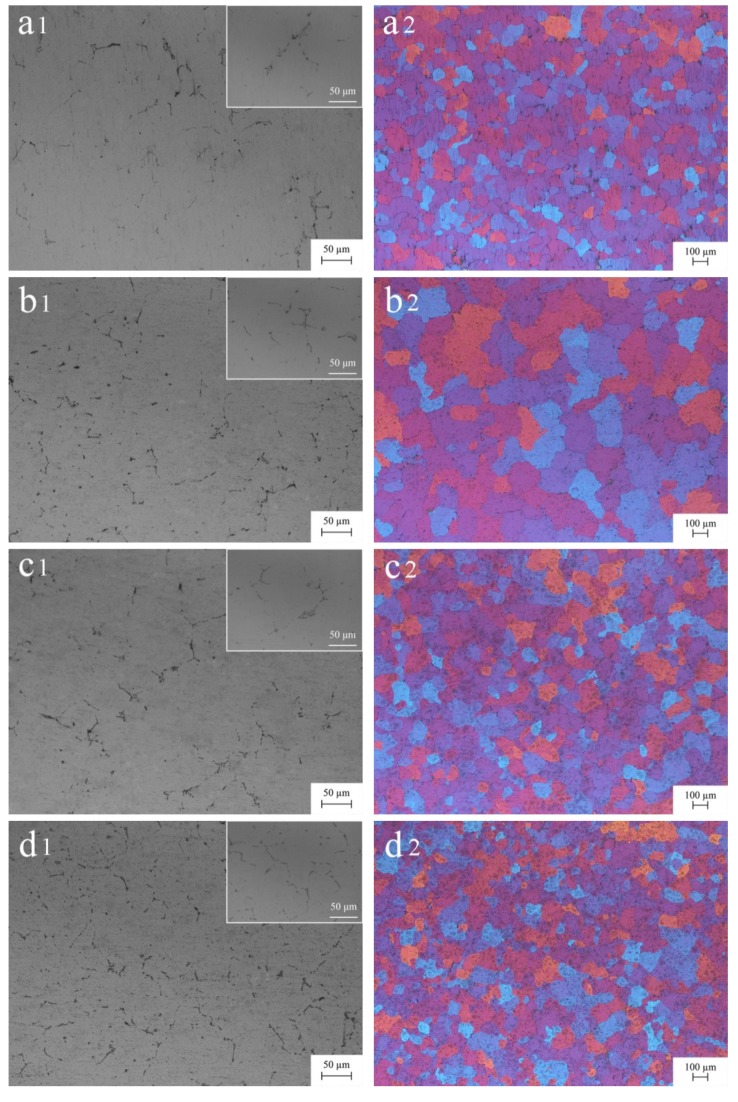

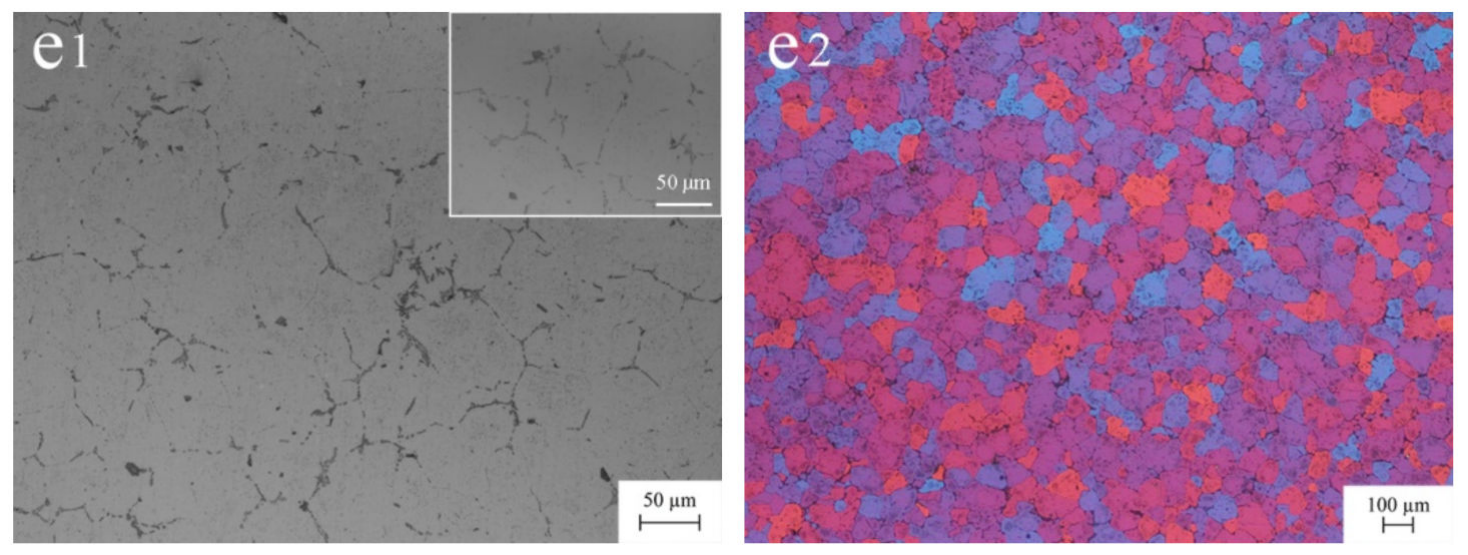
Metallographic structure of homogenized 5052 aluminum alloys with embedded graphs to reflect the local characteristics on the top right of images: (**a1**,**a2**) A0 without Er; (**b1**,**b2**) A1 with 0.1% Er; (**c1**,**c2**) A2 with 0.2% Er; (**d1**,**d2**) A3 with 0.4% Er and (**e1**,**e2**) A4 with 0.8% Er.

Figure 3 shows the metallographic structure of homogenized 5052 aluminum alloys. Compared with the as-cast microstructure, the second phase was mildly refined and distributed more dispersively. This was mainly due to the re-dissolution of the second phase in the homogenization process, which was reflected in the roundness of edge and corner (Figure 2a1–e1; Figure 3a1–e1). Homogenization had little effect on grain size of the 5052 aluminum alloy (A0), but it could improve the uniformity of size distribution (Figure 2a2 and Figure 3a2). For 5052 + Er aluminum alloys (A1–4), homogenization could significantly refine the grain (Figure 2b2–e2; Figure 3b2–e2). Especially when Er content was 0.8%, the grain size was reduced to about 112 μm and the dendrite in the grain was almost eliminated (Figure 2e2 and Figure 3e2). These evidences showed that homogenization could activate Er.

Previous studies showed that the refinement effect of Er on the grain of pure aluminum first appeared when Er content was 0.2%. For the Al-4.5Mg alloy, it should reach 0.4%. When Er content continued to increase, the grain would be further refined. Additionally, the effect of homogenization on grain size could be ignored [23,24]. These results indicated that the refining effect of Er was primarily related to its solid solubility in aluminum alloys. When Er content was low, it mainly existed in the Al matrix in the form of a solid solution, which would not produce a grain refinement effect. When Er content was high, part of Er reacted with Al to form primary Al_3_Er, which increased heterogeneous nucleation sites on the one hand and blocked the movement of the grain boundary on the other hand [25]. In the experiment, only when Er content was 0.4%, the grain size was close to that of A0 (Figure 2a2,d2). Lower or higher than this content, it could not refine the grain, but resulted in coarsening, which maybe had a close relationship with the composition undercooling induced by the addition of Er (Figure 2b2–e2). In particular, the grain size of 5052 + Er aluminum alloys was greatly reduced after homogenization and the size distribution became uniform. When Er content was excessive (0.8%), the effect of grain refinement could be excited violently (Figure 2e2 and Figure 3e2). Considering the melting point of the formed AlEr was much higher than that of aluminum alloys, it could not completely re-dissolve into the Al matrix during homogenization, but only released a small amount of Er through the corner rounding of Er-containing phases (Figure 3e1). Of course, the disappearance of intragranular dendrite also proved this view partly (Figure 3e2). Essentially, homogenization refined the grain by controlling Er content and distribution in the Al matrix. The results of this experiment were quite different from the previous research mostly due to the difference of microstructure caused by the forming method of aluminum alloy ingots with a big width-to-thickness ratio.

**Figure 4:**
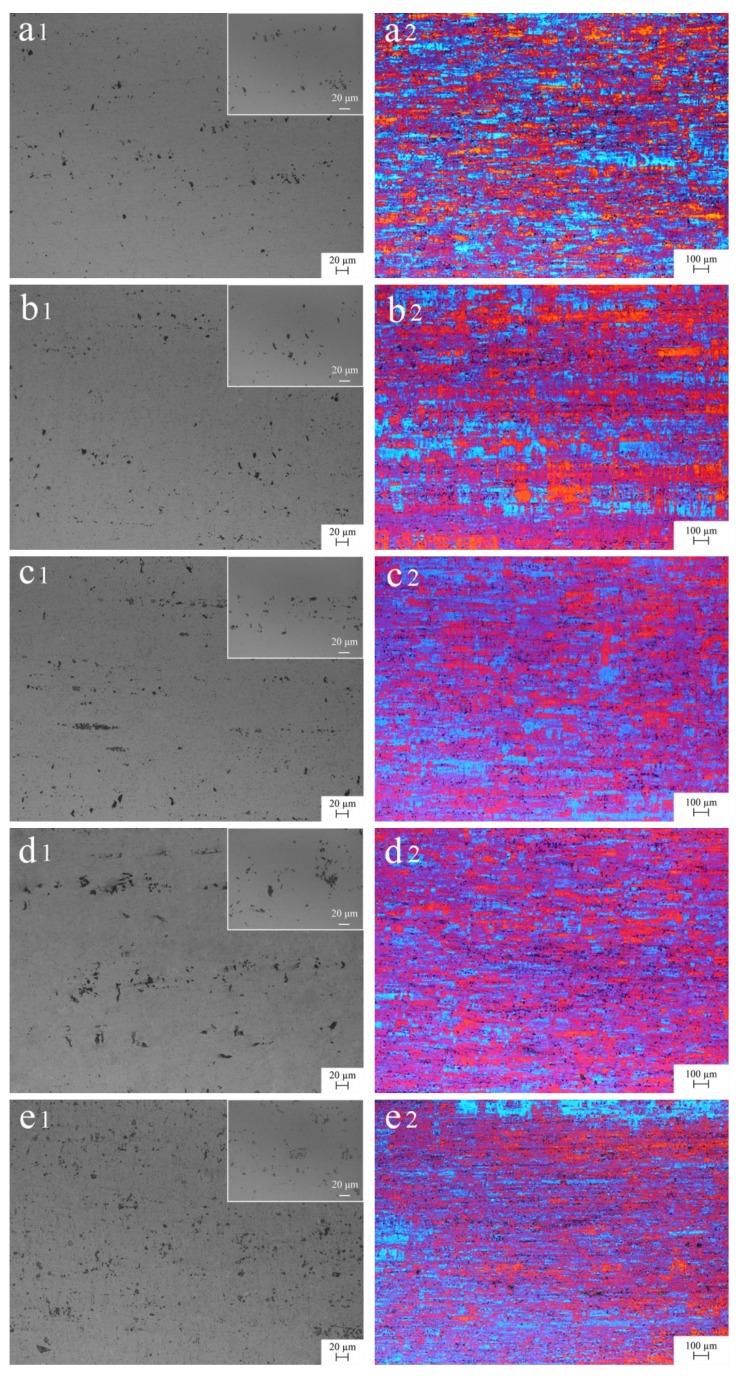
Metallographic structure of cold-rolled 5052 aluminum alloys with embedded graphs to reflect the local characteristics on the top right of images: (**a1**,**a2**) A0 without Er; (**b1**,**b2**) A1 with 0.1% Er; (**c1**,**c2**) A2 with 0.2% Er; (**d1**,**d2**) A3 with 0.4% Er and (**e1**,**e2**) A4 with 0.8% Er.

Figure 4 shows the metallographic structure of cold-rolled 5052 aluminum alloys. After hot rolling and cold rolling, the second phase with a size of approximately 10 μm was elongated or broken along the rolling direction, and evenly distributed in the Al matrix. It could be found that when Er content was 0.1%, the precipitation of the second phase seemed almost the same as that of A0 (Figure 4a1,b1). With the increase of Er content, the distribution of the second phase was more dispersive (Figure 4b1–e1). The grain change was similar to the second phase. Concretely, the fibrous grain of A0 was broken seriously with a length of less than 200 μm and showed regular layered distribution (Figure 4a2). After Er was added, the arrangement of grain did not change. However, the grain boundary was gradually blurred with the increase of Er content, suggesting the deformation degree of grain was increasing (Figure 4b2–e2).

**Figure 5:**
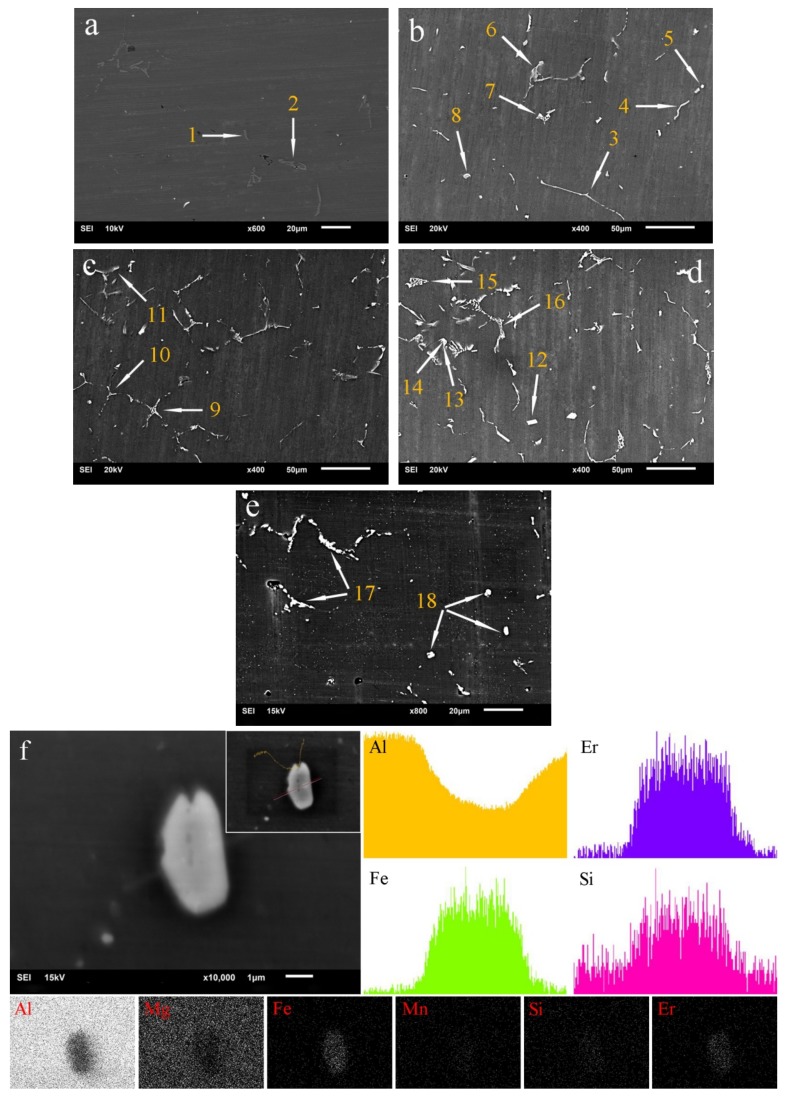
Microstructure of 5052 aluminum alloys observed via SEM: as-cast (**a**) A0 without Er, (**b**) A2 with 0.2% Er, (**c**) A3 with 0.4% Er, (**d**) A4 with 0.8% Er; homogenized (**e**) A3 with 0.4% Er and (**f**) a dot phase analyzed via EDS line scanning and surface scanning.

Figure 5 shows the SEM image of as-cast and homogenized 5052 aluminum alloys. EDS composition analysis was carried out for the second phase with different morphologies in 5052 aluminum alloys, and their types were determined based on relevant literatures [26]. The EDS test results are shown in Table 2. It could be observed that there were two different second phases with a size of 10–20 μm in A0. Both the coarse short-acicular phase (arrow 1) and the irregular phase (arrow 2) were Al_3_Fe, indicating Mg had basically dissolved into the Al matrix (Figure 5a). A2 consisted of six different second phases with various morphologies. Among them, the long-acicular phase (arrow 3), the bent rod phase (arrow 4) and the irregular phase (arrow 6) should be Al_6_Fe, while the dot phase (arrow 5) and the E-type phase (arrow 7,8) should be primary AlEr adsorbing a little Fe or/and Si (Figure 5b). A3 was mainly composed of three different second phases in morphologies of Chinese character (arrow 9), arborization (arrow 10) and feather (arrow 11) with a size of 30–50 μm, all of which were primary AlEr with/without a small amount of Fe or Si (Figure 5c). At this point, the grain refinement began to appear (Figure 2b2–d2). A4 mostly had a variety of second phases with four morphologies. The block phase (arrow 12) was AlCrMg with a little Er. Both the flower-like phase (arrow 15) and the Chinese character phase (arrow 16) were primary AlEr containing no/trace Fe and Si. In particular, the bone phase composition was not uniform, i.e., a large number of Er and a small amount of Si were found at the end of the phase (arrow 14), while only Si was detected at the bone rod (arrow 13; Figure 5d). When casting with the metal model, there would always be component fluctuation and energy fluctuation in molten metal. This was especially true for the preparation of the 5052 aluminum alloy ingots with a big width-to-thickness ratio. In the process of solidification, supersaturated Er and Fe were expelled from grain and accumulated at the front of interface between solid and liquid, promoting the transformation of the dot phase and E-type phase into the Chinese character phase, flower-like phase and so on. After homogenization, the second phase in A3 disintegrated due to re-dissolution, and the size was reduced to 5–20 μm (Figure 5e). The composition analysis of long bar phases (arrow 17) and dot phases (arrow 18) showed that both were AlFeSiEr, but had a different morphology. Meanwhile, it could be found that dot phases here were quite different from those in Figure 5b–d, which was necessary to have an in-depth study. By EDS line scanning, it could be observed that Fe, Er and Si were the main elements in the center of the dot phase on the far right where Al concentration decreased significantly. The EDS surface scanning could also obtain a similar result (Figure 5f). Some studies showed that Er was easy to gather with Fe, Mn and other elements, but it was difficult to form intermetallic compounds. Er had a strong interaction with Al or Si, and the Gibbs free energy of Er_5_Si_3_ precipitation was smaller than that of Al_3_Er, i.e., Er reacted with Si preferentially and the excess would combine with Al to form Al_3_Er [27,28,29]. Generally, in Al–Mg alloys, Fe preferred to react with Al in the early stage of solidification [26]. Therefore, the dot phase was probably a mixture of ErSi, AlEr and AlFe. The morphology and distribution of AlEr in the homogenized structure was obviously different from that in the as-cast structure, which should be secondary. At this time, the grain refinement was more significant Figure 2d2 and Figure 3d2).

### 3.2. Mechanical Properties

**Figure 6:**
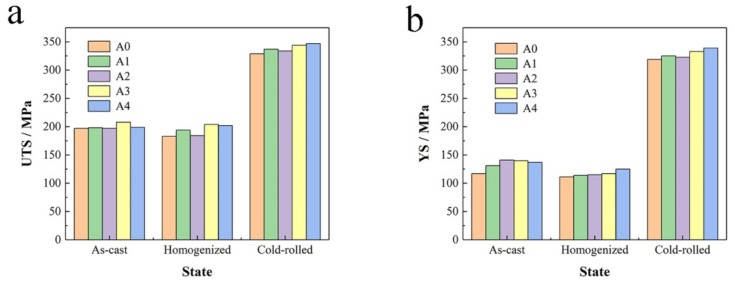

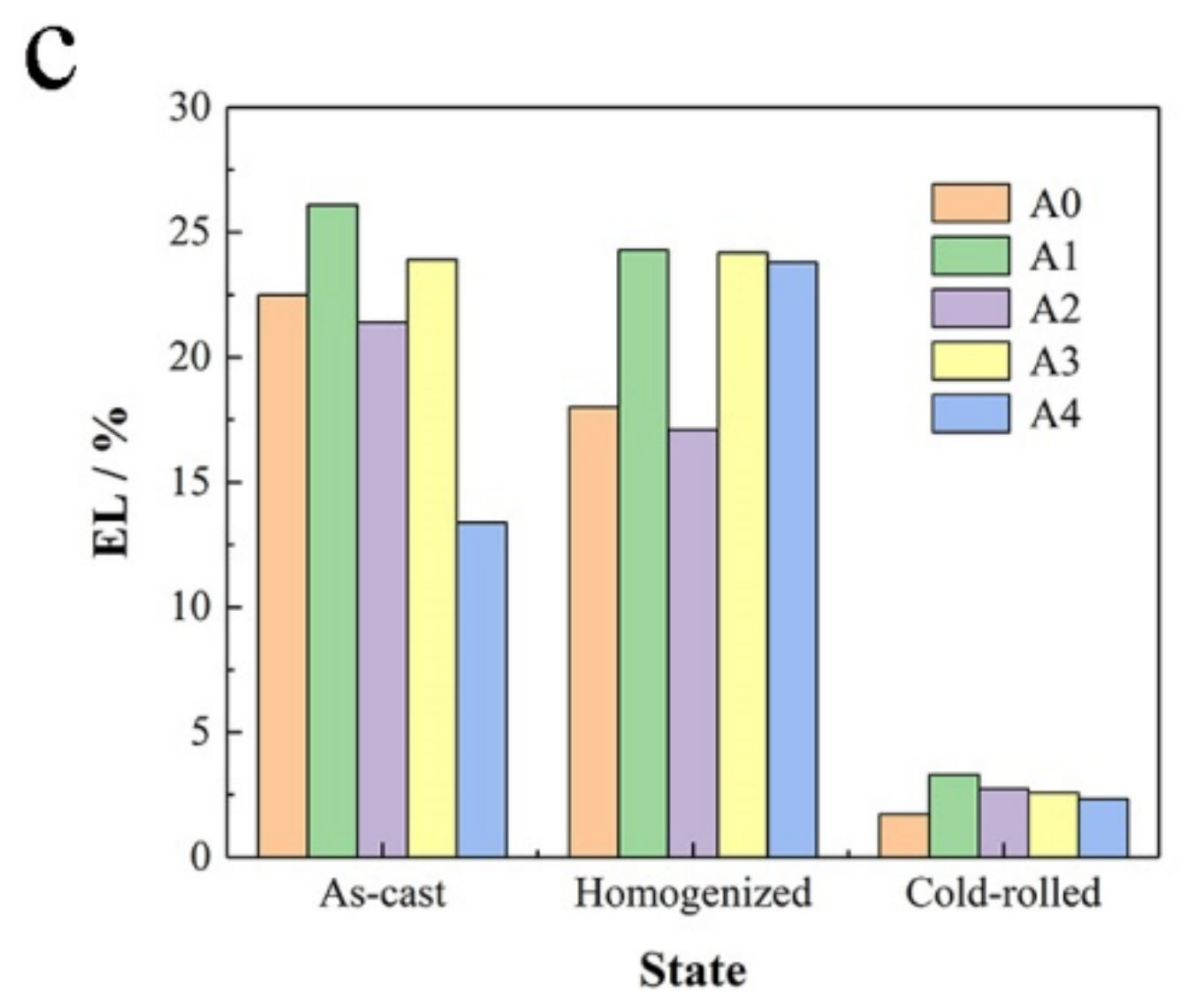
Mechanical properties of 5052 aluminum alloys tested via tensile testing machine: (**a**) UTS; (**b**) YS and (**c**) EL.

Figure 6 shows the mechanical properties of 5052 aluminum alloys. It could be observed that the UTS, YS and EL of as-cast A0 were 197 MPa, 117 MPa and 22.5% respectively. After adding Er, the UTS and YS slightly increased, the highest A3 increased by 5.58% and 19.66%. While, the change of EL fluctuated greatly, the best A1 increased by 16%. It was worth noting that when Er content was 0.8%, the EL decreased by 40.44%, which might be due to the high Er content, resulting in the formation of coarse Er-containing phases and impurities at the grain boundary (Figure 2e1 and Figure 5d). The strength of the 5052 aluminum alloy (A0) and 5052 aluminum alloys with low Er content (A1, A2) would be significantly reduced by long time homogenization at high temperature. However, those with high Er content (A3,A4) would not be affected and their plasticity would be improved. A3 showed a trend of strength decreasing and EL increasing and A4 exhibited an increase in UTS and EL, which were the result of grain refinement and grain growth inhibition caused by the precipitated Er-containing phases (Figure 3d2,e2). After rolling, the strength and EL of 5052 + Er aluminum alloys were higher than those of the 5052 aluminum alloy, especially the EL, increasing by 35.47–92.44%. When Er content increased, the strength increased and the EL decreased. All test results are shown in Table 3.

**Figure 7:**
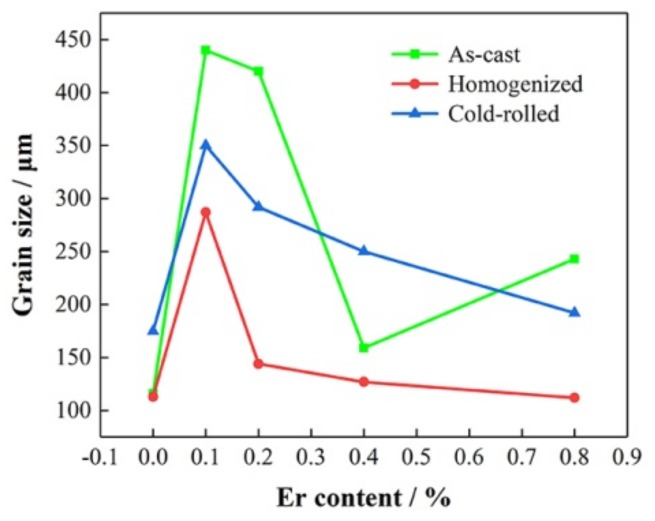
Relation between grain size and Er content of 5052 aluminum alloys.

Figure 7 shows the relation between grain size and Er content of 5052 aluminum alloys. It could be seen that the mechanical properties (especially the UTS and YS) of 5052 aluminum alloys in as-cast and homogenized states were not decreased, but slightly increased, even though the grain became coarsened after adding Er. In fact, the addition of Er could slightly improve the room temperature strength of Al–Mg alloys and the effect was not as obvious as that on the high temperature strength due to the stability of Al_3_Er at elevated temperature [24]. For the 5052 aluminum alloy, it was not only the grain size that could affect the strength, but also the number, size, morphology and distribution of precipitates. It was clear that Er addition helped to refine the second phase, promote the transformation of Al_3_Fe into Al_6_Fe and AlEr with/without a small amount of Fe or Si and make precipitates more dispersive, which were conducive to improving the plasticity. Possibly, the dispersion of the second phase made up for the negative effect of grain coarsening on the strength. The statistical results of grain size are shown in Table 4.

**Figure 8:**
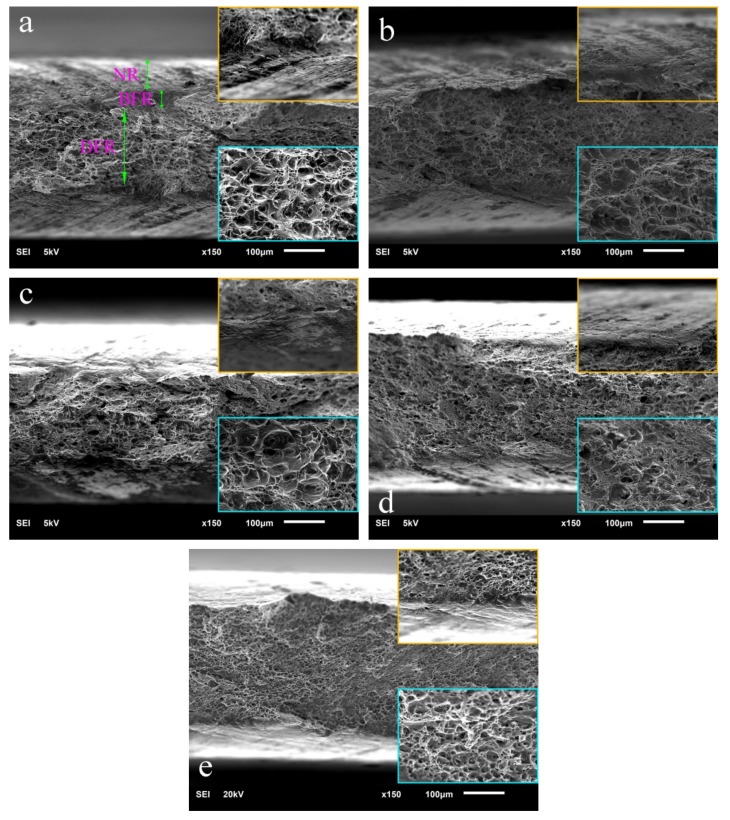
Fracture morphology of cold-rolled 5052 aluminum alloys with embedded graphs to reflect the local characteristics on the right of images observed via SEM: (**a**) A0 without Er; (**b**) A1 with 0.1% Er; (**c**) A2 with 0.2% Er; (**d**) A3 with 0.4% Er and (**e**) A4 with 0.8% Er.

Figure 8 shows the fracture morphology of cold-rolled 5052 aluminum alloys. The fracture section of 5052 aluminum alloys could be divided into three regions from the surface to the center. Taking A0 as an example, the three regions were the necking region (NR), brittle fracture region (BFR) and ductile fracture region (DFR) respectively (Figure 8a). NR was characterized by some parallel sliding steps with an angle of 40 degrees to the transverse direction. BFR mainly presented river patterns. DFR consisted of a large number of dimples. Generally for aluminum alloys, the wider the DFR or NR, and the narrower the BFR were, the better plasticity would be [30,31,32]. When Er was added, the DFR was widened obviously, the NR became flat, and the transition between the adjacent regions got smoother, suggesting the plasticity was improved. With the increase of Er content, the width and the average dimple size of DFR were steadily decreasing, while the width of BFR was slowly increasing, indicating the plasticity was reducing (Figure 8b–e).

## 4. Conclusions

The microstructure and mechanical properties of the 5052 aluminum alloy with a big width-to-thickness ratio in as-cast, homogenized and cold-rolled states were studied by adding trace Er to clarify the grain refinement of Er and the relationship between Er content and alloy strength and plasticity. The following conclusions could be drawn:

(1) The microstructure of the as-cast 5052 aluminum alloy with a big width-to-thickness ratio was mainly composed of α-Al and Al_3_Fe. After adding Er, the second phase was slightly refined, and Al_3_Fe was transformed into Al_6_Fe and AlEr with/without a small amount of Fe or Si. The effect of Er on grain refinement was related to its content. When Er content was lower or higher than 0.4%, the grain would coarsen.
(2) Homogenization had little effect on the grain size of the 5052 aluminum alloy with a big width-to-thickness ratio, but it could improve the uniformity of grain size distribution. Homogenization was conducive to refining the grain by controlling Er content and distribution in the Al matrix. AlFeSiEr with a refining effect might be a mixture of multiple phases blending with each other.
(3) The ultimate tensile strength, yield strength and elongation of the as-cast 5052 aluminum alloy with a big width-to-thickness ratio were 197 MPa, 117 MPa and 22.5% respectively. The strength was the highest, when Er content was 0.4% and the elongation was the best at 0.1% Er content. Long time homogenization at high temperature would significantly reduce the strength of the 5052 aluminum alloy and 5052 aluminum alloys with low Er content, but help to improve the plasticity of those with high Er content.
(4) The fracture morphology of cold-rolled 5052 aluminum alloys with a big width-to-thickness ratio was composed of a necking region, brittle fracture region and ductile fracture region. With the increase of Er content, the width and the average dimple size of DFR were gradually decreasing, while the width of BFR was slowly increasing, which was consistent with the change of elongation.

## Figures and Tables

**Table 1:** Chemical composition of 5052 aluminum alloys (wt. %).

Alloy		Elements								
	Si	Fe	Cu	Mn	Mg	Cr	Zn	Ti	Er	Al
A0	0.052	0.27	0.066	0.072	2.30	0.17	0.070	0.013	0	Other
A1	0.042	0.26	0.070	0.071	2.37	0.16	0.065	0.010	0.1	Other
A2	0.044	0.26	0.066	0.070	2.34	0.16	0.082	0.009	0.2	Other
A3	0.053	0.26	0.064	0.068	2.42	0.18	0.085	0.010	0.4	Other
A4	0.055	0.27	0.065	0.069	2.39	0.17	0.087	0.009	0.8	Other

**Table 2:** EDS results of the second phase of as-cast and homogenized 5052 aluminum alloys (at. %).

Arrow	Elements					
	Al	Fe	Mg	Si	Er	Cr
1	70.86	29.14	-	-	-	-
2	71.24	27.82	0.94	-	-	-
3	86.68	11.85	1.26	-	-	0.21
4	89.00	8.70	1.88	0.42	-	-
5	85.65	-	2.37	1.13	10.85	-
6	90.28	7.99	1.73	-	-	-
7	91.33	0.29	1.56	-	6.82	-
8	91.28	0.41	2.01	0.73	5.57	-
9	92.90	-	2.95	-	4.15	-
10	93.33	0.80	2.82	-	3.05	-
11	91.82	-	3.08	0.85	4.25	-
12	85.38	-	7.25	-	1.92	5.45
13	93.58	-	3.57	2.85	-	-
14	90.88	-	2.29	0.96	5.87	-
15	93.57	-	2.82	-	3.61	-
16	87.51	0.90	1.49	0.88	9.22	-
17	79.27	12.89	0.30	0.81	6.73	-
18	80.21	11.27	1.38	0.82	6.32	-

**Table 3:** Mechanical properties of 5052 aluminum alloys (ultimate tensile strength (UTS), yield strength (YS) MPa and elongation (EL) %).

Alloy	As-Cast			Homogenized			Cold-Rolled		
	UTS	YS	EL	UTS	YS	EL	UTS	YS	EL
A0	197	117	22.5	183	111	18.0	329	319	1.72
A1	198	131	26.1	194	114	24.3	337	325	3.31
A2	197	141	21.4	184	115	17.1	334	323	2.75
A3	208	140	23.9	204	117	24.2	344	333	2.59
A4	199	137	13.4	202	125	23.8	347	339	2.33

**Table 4:** Grain size of 5052 aluminum alloys (μm).

Er Content	Grain Size		
	As-Cast	Homogenized	Cold-Rolled
0	116	113	175
0.1%	440	287	350
0.2%	420	144	292
0.4%	159	127	250
0.8%	243	112	192
